# Short-, Medium-, and Long-Chain Chlorinated Paraffins in Indoor Dust from South China and the Midwestern United States

**DOI:** 10.3390/toxics13060428

**Published:** 2025-05-23

**Authors:** Shuyue Wang, Qiuyan Ke, Wenwen Sun, Yukun Chen, Mehvish Mumtaz, Yumeng Shi, Xiaotu Liu

**Affiliations:** 1 College of Environment and Climate, Guangdong Key Laboratory of Environmental Pollution and Health, Jinan University, Guangzhou 510632, China; wangshuyue@stu2022.jnu.edu.cn (S.W.); keqiuyan0824@163.com (Q.K.); 2 SCIEX (China) Co., Ltd., Guangzhou 510623, China; wenwen.sun@sciex.com (W.S.); yukunchen9@gmail.com (Y.C.); 3 Department of Fisheries and Wildlife, Michigan State University, East Lansing, MI 48824, USA; mumtazme@msu.edu; 4 College of Earth and Environmental Sciences, University of the Punjab, Lahore 54590, Pakistan; 5 MOE Key Laboratory of Pollution Processes and Environmental Criteria, College of Environmental Science and Engineering, Nankai University, Tianjin 300350, China

**Keywords:** chlorinated paraffins, house dust, daily intake, long-chain chlorinated paraffins

## Abstract

In the present study, liquid chromatography–quadrupole time-of-flight mass spectrometry (LC-QTOF-MS) was employed to analyze chlorinated paraffin (CP) homolog distributions and concentrations in household dust from South China and the Midwestern United States. The median levels of short-, medium-, and long-chain CPs (∑SCCPs, ∑MCCPs, ∑LCCPs) in South China were quantified as 23.1, 36.2, and 32.8 μg/g, respectively. Comparatively, the corresponding values in the Midwestern U.S. samples were 9.4, 39.5, and 15.4 μg/g, respectively. Notably, ∑LCCP concentrations in South China significantly exceeded those in the U.S. (*p* < 0.05), while no difference was found for ∑SCCPs and ∑MCCPs. Additionally, very short-chain CPs (C_≤9_) were detected in 13% of samples across both regions. The distribution of CP homologues in the dust samples from the two regions was similar, with C_13_, C_14_, and C_18_ groups as the predominant carbon homologue and Cl_7-8_, Cl_7-9_, and Cl_9-10_ as the predominant chlorine homologue of SCCPs, MCCPs, and LCCPs, respectively. Risk assessment indicated dust ingestion-derived CP intakes for adults and toddlers were 2–5 orders of magnitude below reference doses. However, given other exposure pathways and the combined effects of CP monomers, the potential health risks from exposure via dust should not be underestimated.

## 1. Introduction

Chlorinated paraffins (CPs), a class of synthetic chlorinated hydrocarbons derived from paraffin, typically contain 40–70% chlorine by mass [1]. According to chain length, CPs are categorized into three classes: short-chain (SCCPs, C10-13), medium-chain (MCCPs, C14-17), and long-chain compounds (LCCPs, C ≥ 18) [1]. Owing to their exceptional thermal stability and chemical inertness, these compounds have become industrial high-production-volume chemicals, widely applied as flame retardants in polyvinyl chloride products, plasticizers in sealants/adhesives, and lubricant additives in metalworking fluids [1,2,3,4]. Consequently, extensive industrial production and usage have led to their ubiquitous presence across environmental matrices such as atmospheric particulates, terrestrial sediments, and biota [5,6,7,8]. Human biomonitoring studies further demonstrate pervasive exposure, with CPs detected in placental tissues, blood serum, and breast-milk samples [9,10].

Given the persistence, bioaccumulation, and long-distance mobility, great concern has been raised about the occurrence and pollution of SCCPs in recent years [11,12,13]. The manufacture and application of SCCPs are subject to strict controls in regions such as the U.S., Japan, Canada, and the European Union [5]. In May 2017, the Stockholm Convention classified SCCPs under Annex A as persistent organic pollutants, reinforcing global regulatory measures [14]. Despite the restrictions on SCCPs, the utilization and disposal of products containing them continue to permit their persistence in the environment for extended durations. Concurrently, regulatory limitations on SCCPs have led to increased reliance on MCCPs and LCCPs [15]. A limited number of studies have shown that MCCPs and LCCPs also have certain toxic effects and adverse health effects. For example, Deng et al. demonstrated that LCCP exposure impairs proliferation and autophagy in chicken embryo hepatocytes via oxidative stress [16]. Nevertheless, available environmental monitoring data for LCCPs remain significantly scarcer than for SCCPs.

House dust has been examined as an effective tool for assessing indoor environmental contamination and external human exposure risks [17]. Numerous studies have documented the widespread presence of diverse chemical contaminants in residential dust matrices [18,19]. Recent studies have demonstrated that dust ingestion serves as a significant external exposure pathway to CPs in the general population, ranking second only to dietary intake [5,20]. The occurrence of CPs, especially LCCPs, in house dust is limited. The complexity of mixtures and the difficulty of separation are the main challenges in analyzing CPs. Currently, systematic reports on LCCPs in indoor dust exist only for South Africa, Norway, Japan, and Australia, while data from China and the U.S. remain absent [2,21,22,23].

Recently, we have developed a method based on ultra-performance liquid chromatography (UPLC) coupled to quadrupole time-of-flight mass spectrometry (QTOF-MS) to simultaneously detect SCCPs, MCCPs, and LCCPs in ambient PM_2.5_ [24]. In the present study, we employed this method to measure the concentrations of CPs in house dust collected from South China and the Midwestern U.S. and compared the homologous profiles of CPs in the two regions. We also assessed external exposure of CPs via dust ingestion and dermal absorption. Our study addresses the deficiency of LCCPs in house dust from China and the United States and establishes a foundation for subsequent human health risk assessment.

## 2. Materials and Methods

### 2.1. Chemicals and Reagents

Chlorinated paraffin reference materials were obtained from Ehrenstorfer GmbH (Augsburg, Germany), including SCCP mixtures (C_10-13_; chlorine content: 51.5%, 55.5%, 63%), MCCP mixtures (C_14-17_; 42%, 52%, 57%), and LCCP mixtures (C_18-20_; 36%, 49%). Isotopically labeled standards of ^13^C_10_-trans-chlordane and tert-butyl-paraben-d_9_ were purchased from Cambridge Isotope Laboratories (Andover, MA, USA) and Toronto Research Chemicals (Toronto, Canada), respectively. HPLC-grade solvents were primarily sourced from Oceanpak (Gothenburg, Sweden), with cyclohexane being the exception (Fisher Scientific, Hanover Park, IL, USA). Silica gel (63–100 μm), anhydrous sodium sulfate, and florisil (60–100 mesh) were all obtained from Merck (Whitehouse Station, NJ, USA). Before use, the florisil and silica gel were activated at 130 °C overnight. The acid silica gel (44%) was prepared by thoroughly mixing 44 g concentrated sulfuric acid with 56 g activated silica gel.

### 2.2. Sample Collection

A total of 38 house dust samples in Guangzhou, South China, from 2015 to 2016, and 15 house dust samples in Carbondale, Illinois, U.S., in 2017, were collected (Supplementary Table S1). Floor dust samples from the bedroom and the living room were collected using a commercial vacuum cleaner [25]. A custom-made nylon bag (25 μm pore size) was affixed to the vacuum cleaner nozzle (Electrolux, ZMO1511, 1400 W, Stockholm, Sweden) and vacuumed for 15 to 30 min at each dwelling. Prior to sampling, all the bags were ultrasonically cleaned with HPLC-grade methanol for 30 min. After collection, the nylon bag was removed from the cleaner, wrapped in aluminum foil, placed in a sealed bag, and transported back to the laboratory. The dust samples were removed from the nylon bag and sieved through a 125 μm stainless steel sieve, then stored at −20 °C until analysis. Field blanks were prepared using pre-cleaned sodium sulfate, undergoing identical vacuuming and processing protocols as the dust samples.

### 2.3. Sample Pretreatment and Analysis

Approximately 50 mg of sieved dust was fortified with 5 ng of ^13^C_10_-trans-chlordane as a recovery standard. The spiked samples underwent ultrasonic-assisted extraction using 3 mL of a dichloromethane/n-hexane mixture (1:1, *v*/*v*) for 20 min. After extraction, the supernatant was collected by centrifugation (4000 rpm for 5 min) and then transferred to a clean glass tube, and the extraction process was performed twice. The combined extraction solution was concentrated to 2 mL under gentle nitrogen. A multi-layered chromatographic column (1.5 cm i.d.) was prepared with the following stationary phases (bottom to top): 14 g florisil (deactivated with 1.5% of water), 2 g neutral silica gel, 10 g acid silica gel, and 4 g anhydrous sodium sulfate. Prior to sample loading, the column was pre-conditioned with 50 mL n-hexane to remove potential interferents. The concentrated extract was then introduced onto the column and eluted in two sequential fractions: 60 mL n-hexane and 80 mL n-hexane/dichloromethane (1:1, *v*/*v*). The second fraction was concentrated to near-dryness using rotary evaporation and reconstituted in 100 μL methanol for instrumental analysis.

The analysis of the CPs was conducted using UPLC paired with an X500R QTOF mass spectrometer (AB Sciex, Vaughan, ON, Canada), operating in full-scan mode. Additionally, gas chromatography–triple quadrupole mass spectrometry (GC-MS/MS, 7890B-7000D, Agilent, Santa Clara, CA, USA) was employed to measure the recovery of the ^13^C_10_-trans-chlordane in each sample. Further methodological specifics, including instrumental parameters, are provided in the Supplementary Materials.

### 2.4. Quantification of CPs

The analysis of the CPs was conducted using UPLC-QTOF-MS in negative ion mode ([M − H]^−^), where two diagnostic isotope clusters were monitored as qualitative and quantitative indicators. A total of 36 SCCP, 40 MCCP, and 115 LCCP congeners was used (Supplementary Tables S2 and S3). The identification criteria included a mass accuracy of [M − H]^−^ ions within a 10 ppm error threshold, retention time deviation <±0.2 min from standards, and isotope ratios matching theoretical values within 15% tolerance. The quantification methodology employed the linear correlation between chlorine content and total response factors as established by Reth et al. [26].

### 2.5. Quality Assurance and Quality Control

Prior to use, all the glassware was heated at 450 °C for 6 h. Field blank samples (anhydrous sodium sulfate) were prepared along with the sample collection. Each batch contains ten samples and one laboratory blank sample (50 mg anhydrous sodium sulfate). Trace levels of SCCPs, MCCPs, and LCCPs were identified in procedural and field blanks with a range of 0–13.4, 0–29.2, and 0–9.5 ng/g, respectively. The average recoveries of ^13^C_10_-trans-chlordane were 89% ± 21%. The limit of quantification (LOQs) of ∑SCCPs, ∑MCCPs, and ∑LCCPs were 15, 31, and 10 ng/g, respectively, calculated as ten times the standard deviation of procedural blanks.

### 2.6. Exposure Assessment

We estimated the daily intake (EDI, ng/kg BW/day) and hazard quotient (HQ, unitless) of CPs for adults and toddlers via ingestion and dermal absorption of dust-associated CPs. The EDI and HQ were determined using the equations as follows [20]:

$${\mathrm{EDI}}_{\mathrm{ingestion}}=\frac{C_{i}\times \mathrm{DIR}\times \mathrm{EF}}{\mathrm{BW}}\times \mathrm{BA}$$

$${\mathrm{EDI}}_{\mathrm{dermal adsorption}}=\frac{C_{i}\times \mathrm{BSA}\times \mathrm{AS}\times \mathrm{AF}\times \mathrm{EF}}{\mathrm{BW}}\times \mathrm{BA}$$

$${\mathrm{EDI}}_{\mathrm{Total}}=({\mathrm{EDI}}_{\mathrm{ingestion}}+{\mathrm{EDI}}_{\mathrm{dermal adsorption}})$$

$$\mathrm{HQ}=\frac{{\mathrm{EDI}}_{\mathrm{Total}}}{\mathrm{TDI}}$$

where DIR represents the dust ingestion rate (g/day); C_i_ is the concentration of CPs in house dust (ng/g); EF is the proportion of daily time spent in residential environments (unitless); BA is the bioaccessibility of CPs (unitless); BW is body weight (kg); BSA is body surface area (m^2^); AS represents the adhesion rate of dust on the skin (g/m^2^); AF is the adsorption rate of CPs in the skin (unitless); and TDI is the tolerable daily intake (μg/kg/day). The TDI of SCCPs, MCCPs, and LCCPs was uniformly adopted as 100 μg/kg/day [27], and the values of the other parameters used in this study are summarized in Table S4.

### 2.7. Data Analysis

The reported concentrations were recovery-adjusted using surrogate standards and corrected for procedural blanks. Compounds detected in >60% of samples underwent statistical analysis, with measurements below the limit of quantification (LOQ) substituted by LOQ/√2. Intergroup correlations were assessed using Spearman’s rank test, while Mann–Whitney U tests compared CP concentrations between the two regions. All analyses were performed in SPSS V2.0 (PASW Statistics 18.0, IBM Inc., Armonk, NY, USA), with significance set at α = 0.05.

## 3. Results and Discussion

### 3.1. Concentration of CPs in House Dust

SCCPs, MCCPs, and LCCPs with at least one congener were detected in all of the indoor dust samples from South China and the Midwestern U.S. Notably, congeners with very short carbon chains (vSCCPs, C_≤9_) were detected in five China and two U.S. dust samples with a detection rate of 13%, and very long-chain CPs (vLCCPs, C_≥20_) were also detected in all samples. The total concentrations of CPs with C_9_ to C_35_ ranged from 22.2 to 466 μg/g (median: 100 μg/g) and 15.1 to 246 μg/g (median: 82.9 μg/g) in dust from South China and the Midwestern U.S., respectively (Table 1). Compared with other industrial chemicals widely reported in indoor dust, the concentration of CPs was lower than phthalates (median: 601 μg/g) [28], while much higher than that of organophosphate esters (median: 7.5 μg/g) [29] and polybrominated diphenyl ethers (median: 0.8 μg/g) [30]. The high concentrations and wide detection reflect the broad applications of CPs in household products, raising great attention to their ultimate destinations and subsequent risks to humans.

The median concentrations of ∑SCCPs, ∑MCCP, and ∑LCCPs in house dust were 23.1, 36.2, and 32.8 μg/g from South China and 9.36, 39.5, and 15.4 μg/g from the Midwestern U.S., respectively. Notably, ∑MCCP concentrations dominated in both regions, exceeding ∑SCCP levels in South China and surpassing both ∑SCCP and ∑LCCP concentrations in Midwestern U.S. samples (*p* < 0.05). This MCCP predominance aligns with global accumulative production patterns, where MCCPs constitute 57% of total CPs, compared to 28% for SCCPs and 15% for LCCPs [31]. However, the concentrations of ∑LCCPs substantially exceeded those of ∑SCCPs in dust from South China. This may be related to the use of some products that may contain LCCPs in households in Southern China. Another reason might be that our sampling area of South China has a subtropical climate with high temperatures, and SCCPs with lower octanol–air partition coefficients are more likely to volatilize into the gas phase. The concentrations of ∑LCCPs in house dust from South China were significantly higher than those from the Midwestern U.S. (*p* < 0.05). The same trend was observed for SCCPs, but the results were not significant, while no difference was observed for ∑MCCPs. The variations in production and consumption of CP products and related policies between the two countries may primarily account for the disparity of CPs in house dust between both locations. China’s dominant position in the CP market inevitably leads to substantial releases and resulted in relatively high levels of CPs in environmental media [31].

Numerous studies have documented the concentration of SCCPs and MCCPs in indoor dust (Table S5). South China’s house dust showed notably elevated ∑SCCP concentrations (median: 23.1 μg/g) compared to the Midwestern U.S. (9.36 μg/g), Norway (5.8 μg/g), and Australia (9.4 μg/g) but aligned with levels observed in South Africa (16 μg/g) and several Chinese cities (Harbin: 47.2 μg/g; Beijing: 98.7 μg/g; Qingyuan: 46.5 μg/g) [2,4,5,20,21]. The concentrations of ∑MCCPs in house dust from South China (median: 36.2 μg/g) and the Midwestern U.S. (median: 39.5 μg/g) were comparable to those found in house dust from Oslo, Norway (median: 21 μg/g); Pretoria, South Africa (median: 46 μg/g); and Australia (median: 95 μg/g) [2,21,22]. Although studies on LCCPs are relatively limited than on SCCPs and MCCPs (Table S4), our data were comparable to the concentration of LCCPs in house dust from Oslo, Norway (median: 8.1 μg/g); Pretoria, South Africa (median: 11 μg/g); Colombia (median: 13 μg/g); and Thailand (median: 12 μg/g) [2,22,23].

Strong inter-correlations emerged among ∑SCCPs, ∑MCCPs, and ∑LCCPs across both regions (Table S6), indicating potential shared sources and environmental behaviors among these structural analogs. The most robust relationships occurred between ∑SCCPs and ∑MCCPs in South China (Spearman Coefficient = 0.717, *p* < 0.001), and ∑MCCPs and ∑LCCPs in both South China (Spearman Coefficient = 0.726) and the Midwest U.S. (Spearman Coefficient = 0.832) (all *p* < 0.001). Weaker but statistically significant correlations were found for ∑SCCPs-LCCPs pairs in both locations, along with ∑SCCPs-MCCPs in the Midwest U.S. These observed correlation patterns align with current industrial practices where commercial CP formulations typically contain overlapping mixtures of SCCP, MCCP, and LCCP components with varying carbon chain lengths [32]. Furthermore, environmental transformation processes, including microbial degradation, oxidative reactions, combustion, and photolytic breakdown, could induce carbon chain shortening in higher molecular weight CPs, potentially generating SCCPs as degradation byproducts [7,33,34]. This degradation pathway might contribute to the observed strong correlations, particularly in environmental matrices like dust that accumulate CPs from both direct emissions and transformation.

### 3.2. Homologue Profiles of CPs in House Dust

**Figure 1:**
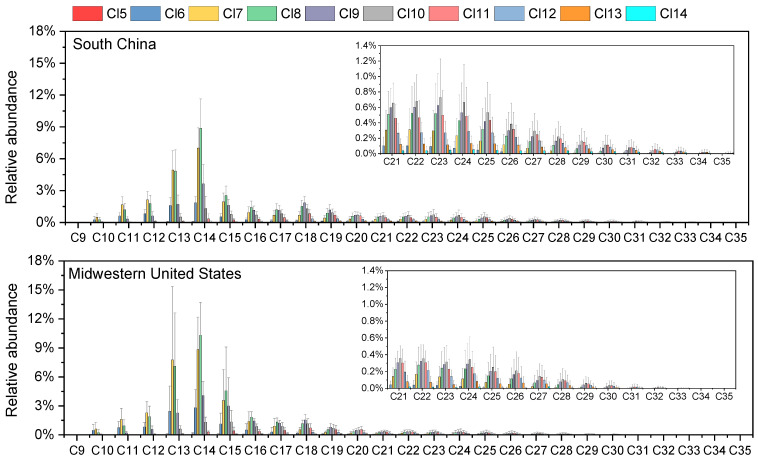
Mean relative abundance with standard deviation error bars of CP homologues in house dust samples from South China and Midwestern U.S.

The homologue distribution patterns of SCCPs, MCCPs, and LCCPs showed remarkable consistency between South China and Midwestern U.S. house dust samples (Figure 1). Across both regions, C13 emerged as the predominant chain length for SCCPs, accounting for the highest proportion, with successively lower abundances observed for C12, C11, and C10 congeners. The carbon group profiles of SCCPs for house dust from South China and the Midwestern U.S. were similar to those found in dust samples from most previous studies [2,21,22,35]. Among MCCPs, C_14_-CP groups were the dominant carbon homologue profiles in the two study regions, followed by C_15_, C_16_, and C_17_. A similar homologous trend was also found in Belgian indoor dust [36], ambient PM2.5 from South China [24], and Norway handwipe samples [37]. The C_14_-CP groups have been used in most commercial MCCP products with more than 60 wt% in the U.S. [38]. Similarly, CP-52, a commercial mixture widely used in China, also uses the C_14_-CP congener group as the main component [39,40]. This explains the predominance of C_14_ in house dust from both regions. However, the proportions of C15 in house dust from the Midwest U.S. (21.7 ± 3.0%) were significantly higher than those from South China (19.1 ± 4.3%) (Figure S1). These differences imply region-specific influencing factors of CP carbon compositions.

Within LCCPs, C_18_-CP groups were the dominant carbon homologue profiles of LCCPs in most of the dust samples from South China and the Midwest U.S., followed by C_19_ and C_20_. However, the carbon chain length distribution of LCCPs exhibited more variability compared to SCCPs and MCCPs in the dust samples. C22 to C25 groups were the dominant profiles, accounting for more than 50% of the total LCCPs in three dust samples from China and two samples from the Midwest U.S. Remarkably, carbon chain lengths of C28 to C35-CP were also detected in most samples from South China and a few from the Midwest U.S. (Figure S2). Analytical data from technical mixtures reveal substantial compositional variations among commercial formulations [41]. For example, analysis revealed that C_23_-CP homolog groups showed the highest abundance in commercial CP-70 formulations [13], while C_18_-CP groups were the dominant carbon homologue profiles of LCCPs in the commercial mixture of CP-52 [15]. This observed variability in homolog distribution patterns likely stems from the coexistence of multiple industrial formulations containing distinct carbon chain profiles.

The distribution profiles of Cl groups were generally consistent between samples from the two regions (Figure 1). In SCCPs, Cl_7_ constituted the most abundant chlorination level, with Cl_8_ and Cl_6_ present as secondary components. MCCPs exhibited a distinct profile dominated by Cl_8_ homologues, while Cl_7_ and Cl_9_ showed lower abundance. For LCCPs, Cl_9_, Cl_10_, and Cl_11_ formed the primary homologues. Our chlorine homologues differed from those in indoor dust from South Africa, Australia, and Norway [2,21,22]. The results stem from various countries employing distinct CP mixes of commercial items. The chlorine homologues were directly related to the total chlorine degree in CPs. The degree of chlorination in dust from both Guangzhou and the Midwestern U.S. exhibits a consistent pattern: SCCPs > MCCPs > LCCPs (Table 1). This trend was similar to that observed in South China ambient PM_2.5_, Australia sewage sludge, and Yangtze River Delta wildlife [15,24,42].

### 3.3. Human Exposure via Dust Intake

**Figure 2:**
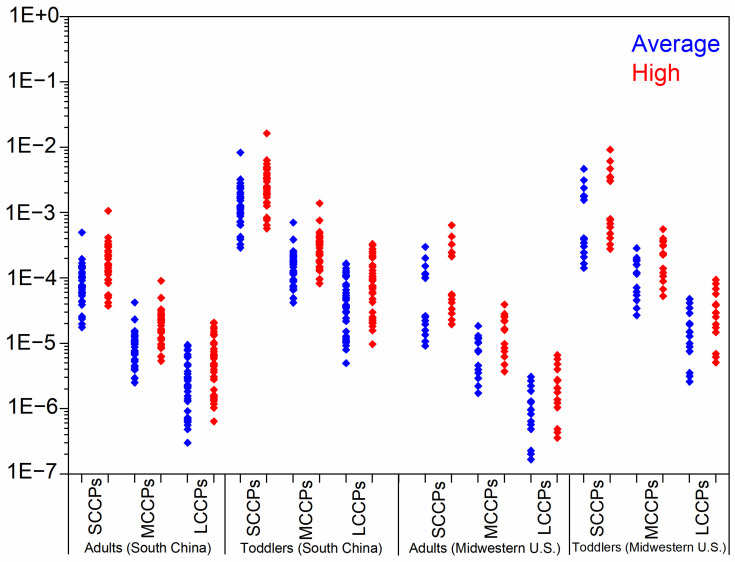
The hazard quotient for toddlers and adults from South China and Midwestern U.S. exposure to SCCPs, MCCPs, and LCCPs under average- and high-exposure scenarios.

We determined the EDIs and HQs of CPs via dust ingestion and derma absorption for both toddlers and adults (Table 2 and Figure 2). In South China, toddlers showed median total EDI values of 12.2 (SCCPs), 12.9 (MCCPs), and 3.83 ng/kg bw/day (LCCPs) under average exposure conditions, increasing to 23.9, 25.3, and 7.53 ng/kg bw/day, respectively, in high-exposure scenarios. Midwestern U.S. toddlers exhibited lower SCCP exposure (4.08 ng/kg bw/day) but comparable MCCP levels (11.6 ng/kg bw/day) under average conditions, with all values approximately doubling in high-exposure situations (Table 2). The EDIs of CPs for toddlers were significantly greater than those for adults, indicating that children are highly exposed to CPs due to increased dust intake rates, prolonged indoor time, and lower body weight. As for the two exposure pathways, dust ingestion dominated exposure pathways, contributing 76–96% of total EDIs under average conditions, with even greater predominance in high-exposure scenarios (Figure S3). These results indicated that ingestion was the dominant way for general populations to be exposed to CPs. However, the exposure to CPs through dermal permeation cannot be ignored.

As shown in Figure 2, the highest HQ of SCCPs, MCCPs, and LCCPs for toddlers and adults in both regions were orders lower than 1. These results imply that indoor dust-related CP exposure cannot cause potential health risks for the general population of South China and the U.S. However, several limitations of our exposure assessments should be mentioned. Firstly, the predicted exposure only aggregates the three chemical types, disregarding the potential mixing effects arising from several isomers and their varying modes of hazardous action and thresholds. The “mixed” effect may lead to different exposure risks. Secondly, although dust ingestion via hand-to-mouth contact may represent a significant exposure pathway of CPs for toddlers [20], other exposure pathways (such as diet, inhalation) also made contributions. For adult populations, alternative exposure pathways may outweigh dust ingestion in significance. An assessment of CP exposure among the general population in Beijing showed that adults were exposed to SCCPs and MCCPs mainly via diet (accounting for 88% and 93% of the total exposure) [20]. Omitting these exposure pathways in our estimation may substantially underestimate the actual risk. Thirdly, although we considered the bioaccessibility of CPs entering the human body via dust ingestion in our assessment, these values were derived from an in vitro study and may be influenced by numerous other factors [43].

## 4. Conclusions

We systematically characterized the concentration and profiles of SCCPs, MCCPs, and LCCPs in residential dust samples from China and the United States. Our findings revealed MCCPs as the dominant congener class in both regions, with LCCPs demonstrating comparable or even higher abundance than SCCPs in South China samples. This pattern highlights the widespread presence of LCCPs in indoor environments and underscores their significance in exposure assessments. We observed consistent homologue distribution patterns between the two geographically distinct regions. We also identified the CP class known as vSCCPs in a few samples from both regions, while very long-chain LCCPs with a carbon chain length up to 35 were also frequently detected. Although our data indicate that exposure to CPs via dust ingestion and dermal absorption is relatively low in abundance, due to the complexity of human exposure pathways, a better elucidation of the exposure risks and relative contribution to CPs through different pathways across multiple environmental matrices (e.g., water, air, food) is needed in future investigations. Furthermore, integrating these data with internal exposure levels measured in human biological samples is also essential for accurate risk evaluation of CP exposure.

## Figures and Tables

**Table 1:** Detection frequencies (DFs), total concentrations and chlorine content of SCCPs, MCCPs, and LCCPs (µg/g) in house dust from South China and Midwestern U.S.

		DF (%)	Total Concentration (μg/g)			Chlorine Content (%)
			Min	Median	Max	
South China	SCCPs	100	5.51	23.1	158	61
	MCCPs	100	11.8	36.2	198	58
	LCCPs	100	4.22	32.8	145	53
	∑CPs		22.2	100	466	
Midwestern U.S.	SCCPs	100	3.27	9.36	107	60
	MCCPs	100	9.15	39.5	97.2	55
	LCCPs	100	2.68	15.4	49.5	50
	∑CPs		15.1	82.9	246	

**Table 2:** Estimated daily intake (ng/kg bw/day) via dust ingestion and dermal absorption of SCCPs, MCCPs, and LCCPs by toddlers and adults under average- and high-exposure scenarios.

		South China			Midwestern U.S.		
		SCCPs	MCCPs	LCCPs	SCCPs	MCCPs	LCCPs
Adults							
Average	min	0.18	0.25	0.03	0.09	0.17	0.02
	median	0.74	0.77	0.23	0.26	0.75	0.10
	max	4.98	4.24	0.95	3.00	1.84	0.31
High	min	0.37	0.54	0.06	0.20	0.37	0.04
	median	1.57	1.66	0.49	0.56	1.60	0.21
	max	10.7	9.07	2.08	6.43	3.94	0.66
Toddlers							
Average	min	2.91	4.19	0.50	1.42	2.69	0.26
	median	12.2	12.9	3.83	4.08	11.6	1.49
	max	83.3	70.7	16.8	46.7	28.6	4.79
High	min	5.71	8.23	0.97	2.79	5.28	0.51
	median	23.9	25.3	7.53	8.01	22.8	2.92
	max	163	139	33.2	91.8	56.2	9.41

## Data Availability

The data supporting the findings of this study are included within the paper and Supplementary Material and are available from the corresponding authors on request.

## Supplementary Materials

The following supporting information can be downloaded at: https://www.mdpi.com/article/10.3390/toxics13060428/s1, Table S1: Instrument methods, Information of sampled dwellings and indoor temperature and humidity data during sampling; Table S2: Quantification and qualification ions of SCCPs and MCCPs; Table S3: Quantification and qualification ions of LCCPs; Table S4: Parameters used for the estimation of daily intake of CPs; Table S5: Concentration of CPs in indoor dust from different regions worldwide; Table S6: Spearman correlation of SCCPs, MCCPs and LCCPs in house dust from South China and Midwestern U.S.; Figure S1: The mean relative abundance of carbon homologues of SCCPs and MCCPs in house dust from South China and Midwestern U.S.; Figure S2: The mean relative abundance of carbon homologues of LCCPs in house dust from South China and Midwestern U.S.; Figure S3: Contribution of estimated daily intakes via dermal exposure and ingestion of dust in toddlers and adults from South China and Midwestern U.S. under different exposure scenarios [44,45,46,47,48,49].
