# Supplementary material for: Short-, Medium-, and Long-Chain Chlorinated Paraffins in Indoor Dust from South China and the Midwestern United States
Source: Toxics. 2025 May 23;13(6):428. doi: 10.3390/toxics13060428 (PMC12197769; doi:10.3390/toxics13060428)
Supplement: Supplementary file 1 [file toxics-13-00428-s001.zip › toxics-3605461-supplementary.pdf]

Short-, Medium-, and Long-Chain Chlorinated Paraffins in Indoor Dust from South China and the Midwestern United States

Shuyue Wang [1], Qiuyan Ke [1], Wenwen Sun [2], Yukun Chen [2], Mehvish Mumtaz [3,4], Yumeng Shi [5,*] and Xiaotu Liu [1,*]

[1] College of Environment and Climate, Guangdong Key Laboratory of Environmental Pollution and Health, Jinan University, Guangzhou 510632, China; wangshuyue@stu2022.jnu.edu.cn (S.W.); keqiuyan0824@163.com (Q.K.)
[2] SCIEX (China) Co., Ltd., Guangzhou 510623, China; wenwen.sun@sciex.com (W.S.); yukunchen9@gmail.com (Y.C.)
[3] Department of Fisheries and Wildlife, Michigan State University, East Lansing, MI 48824, USA; mumtazme@msu.edu
[4] College of Earth and Environmental Sciences, University of the Punjab, Lahore 54590, Pakistan
[5] MOE Key Laboratory of Pollution Processes and Environmental Criteria, College of Environmental Science and Engineering, Nankai University, Tianjin 300350, China
* Correspondence: shiym0519@163.com (Y.S.); liuxiaotu@jnu.edu.cn (X.L.)

Text S1. Instrument methods

The gas chromatography coupled with triple quadrupole mass spectrometer (GC-ENCI-LRMS, Agilent 7890B-7000D) was used to analyze the recovery of $^{13}C_{10}$-trans-chlordane. The mode of ion source was electron capture negative chemical ionization (NCI) source with a 30 m DB-5MS (0.25 mm×0.25 μm) column. Helium was used as carrier gas, and the constant flow was 1.0 mL/min. Methane gas was the reaction gas with a flow rate of 0.4 mL/min. The injection volume was 1 μL. The temperature of the injector, transfer line and ion source were 250℃, 275℃ and 160℃, respectively. The oven temperature rising procedure was as follows: the initial temperature at 100 ℃, 1 min later, raised to 160 ℃ at 30℃/min for 5 min, and finally raised to 310 ℃ at 30 ℃/min for 10 min. Selective ion detection (SIM) mode was used to collect the characteristic ions.

High performance liquid chromatography coupled with electron spray ionization quadrupole time-of-flight mass spectrometry (UPLC-ESI-QTOFMS, AB Sciex X500R) was used to analyze SCCPs, MCCPs and LCCPs in house dust from South China. XBridge ® BEH-C_{18} (2.1×50 mm, waters, USA) was used to separate all samples. The temperature of column was maintained at 40 °C, the injection volume was 10 μL. The mobile phase consisted of 10 mM ammonium acetate aqueous solution (A) and methanol (B) with a flow rate of 0.4 mL/min. The elution procedure was as follows: the initial proportion of mobile phase B was 40% and kept for 0.5 min, rise to 100% by 2 min and kept for 1.5 min, then reduced to 40% by 0.5 min and maintained for 3.5 min. The condition of ESI ion source was as follows: the Curtain gas, Ion source gas 1 and Ion source gas 2 were 30, 55 and 55 psi; source temperature was at 600℃; Ionspray voltage, Declustering Potential and Collision energy were 4500 V, 80 V and 10 V; two reference ions (m/z 616.3550 and 792.4598) were set as internally calibrated ions, the mass accuracy lower than 5 ppm; the analysis was performed in full-scan mode, $[M-H]^-$ was selected as the quantitative ion, and the collection range was 180 to 1200 m/z.

Table S1. Information of sampled dwellings and indoor temperature and humidity data during sampling

	Household Sampling ID	Housing Type	Dwelling Area (m²)	Temperature/°C	Humidity/%
Carbondale, Illinois, U.S.	17US001	Detached Residence	162	25	57
	17US002	Detached Residence	149	25	55
	17US003	Detached Residence	119	26	48
	17US004	Detached Residence	167	25	52
	17US005	Detached Residence	186	25	53
	17US006	Detached Residence	154	27	56
	17US007	Detached Residence	329	28	39
	17US008	Detached Residence	163	26	42
	17US009	Detached Residence	345	26	51
	17US010	Detached Residence	144	27	45
	17US011	Detached Residence	214	25	56
	17US012	Detached Residence	314	27	47
	17US013	Detached Residence	181	26	47
	17US014	Detached Residence	216	28	32
	17US015	Detached Residence	148	25	50
Guangzhou, Guangdong, China	15DW001	High-rise Residential	81	16.2	30
	15DW015	High-rise Residential	130	15.6	77
	16DW031	High-rise Residential	100	29.8	64
	15DW014	High-rise Residential	160	16.9	49
	16DWO26	High-rise Residential	98	28.2	54
	15DW028	High-rise Residential	90	29.2	42
	15DW033	High-rise Residential	110	18.7	45
	15DW048	High-rise Residential	81	16.2	30
	15DW046	High-rise Residential	73	19.3	46
	15DW039	High-rise Residential	82	18.2	57
	16DWO30	High-rise Residential	90	28.2	79
	15DW034	High-rise Residential	116	20.6	66
	16DW013	High-rise Residential	70	26.3	64
	16DW014	High-rise Residential	88	21.2	53
	16DW015	High-rise Residential	95	26.7	51
	16DW016	High-rise Residential	85	29.4	74
	15DW005	High-rise Residential	130	15.6	77
	15DW036	High-rise Residential	109	17.1	73
	15DW007	High-rise Residential	75	16.4	42
	15DW024	High-rise Residential	90	NA	NA

16DWO27	High-rise Residential	100	29.9	80
15DW010	High-rise Residential	90	18.5	47
15DW017	High-rise Residential	128	18.5	86
15DW002	High-rise Residential	73	19.3	46
15DW054	High-rise Residential	98	28.2	54
15DW038	High-rise Residential	95	17.2	59
15DW026	High-rise Residential	128	27.8	56
15DW031	High-rise Residential	90	24	55
15DW047	High-rise Residential	108	18.4	42
15DW044	High-rise Residential	75	18.6	68
15DW037	High-rise Residential	84	18.1	68
15DW020	High-rise Residential	104	24.4	89
15DW009	Apartment, Floor 5	20	19.3	43
15DW003	High-rise Residential	88	20.5	56
16DW001	High-rise Residential	104	18.9	43
16DW002	High-rise Residential	78	21.2	55
16DW003	High-rise Residential	128	26.5	87
16DW004	High-rise Residential	138	24.5	75

Table S2. Quantification and qualification ions of SCCPs and MCCPs

SCCPs	Quantification ion	Qualification ion	MCCPs	Quantification ion	Qualification ion
C9H16Cl4	264.9904	262.9933	C14H26Cl4	333.0710	335.0681
C9H15Cl5	298.9514	300.9485	C14H25Cl5	369.0291	371.0271
C9H14Cl6	332.9124	334.9095	C14H24Cl6	402.9907	404.9879
C9H13Cl7	366.8734	368.8705	C14H23Cl7	436.9518	438.9489
C9H12Cl8	402.8315	400.8345	C14H22Cl8	472.9099	470.9128
C9H11Cl9	436.7926	434.7952	C14H21Cl9	506.8709	504.8738
C9H10Cl10	470.7536	472.7506	C14H20Cl10	540.8319	542.8290
C9H9Cl11	504.7146	506.7117	C14H19Cl11	574.7923	576.7894
C10H18Cl4	277.0084	279.0055	C14H18Cl12	608.7534	610.7504
C10H17Cl5	312.9665	314.9636	C14H17Cl13	642.7144	644.7114
C10H16Cl6	346.9281	344.9310	C15H28Cl4	347.0867	349.0837
C10H15Cl7	380.8891	382.8862	C15H27Cl5	383.0447	385.0418
C10H14Cl8	416.8472	414.8502	C15H26Cl6	417.0064	419.0035
C10H13Cl9	450.8083	448.8112	C15H25Cl7	450.9674	452.9645
C10H12Cl10	484.7693	486.7664	C15H24Cl8	486.9256	484.9284
C10H11Cl11	518.7297	520.7268	C15H23Cl9	520.8866	518.8895
C10H10Cl12	552.6908	554.6878	C15H22Cl10	554.8476	556.8447
C11H20Cl4	291.0241	293.0211	C15H21Cl11	588.8080	590.8050
C11H19Cl5	326.9822	328.9792	C15H20Cl12	622.7690	624.7661
C11H18Cl6	360.9438	362.9409	C15H19Cl13	656.7300	658.7271
C11H17Cl7	394.9048	396.9019	C16H30Cl4	361.1023	363.0994
C11H16Cl8	430.8629	428.8658	C16H29Cl5	397.0604	399.0574
C11H15Cl9	464.8239	462.8268	C16H28Cl6	431.0221	433.0192
C11H14Cl10	498.7849	500.7820	C16H27Cl7	464.9831	466.9802
C11H13Cl11	532.7454	534.7424	C16H26Cl8	500.9412	498.9441
C11H12Cl12	566.7064	568.7035	C16H25Cl9	534.9022	532.9051
C12H22Cl4	305.0397	307.0368	C16H24Cl10	568.8632	570.8604
C12H21Cl5	340.9978	342.9949	C16H23Cl11	602.8236	604.8207
C12H20Cl6	374.9594	376.9565	C16H22Cl12	636.7847	638.7817
C12H19Cl7	408.9205	410.9175	C16H21Cl13	670.7457	672.7427
C12H18Cl8	444.8786	442.8815	C17H32Cl4	375.1180	377.1150
C12H17Cl9	478.8396	476.8425	C17H31Cl5	411.0761	413.0731
C12H16Cl10	512.8006	514.7977	C17H30Cl6	445.0377	447.0349
C12H15Cl11	546.7610	548.7581	C17H29Cl7	478.9987	480.9959
C12H14Cl12	580.7221	582.7191	C17H28Cl8	514.9569	512.9598
C13H24Cl4	319.0554	321.0524	C17H27Cl9	548.9179	546.9208
C13H23Cl5	355.0135	357.0105	C17H26Cl10	582.8789	584.876
C13H22Cl6	388.9751	390.9722	C17H25Cl11	616.8393	618.8363
C13H21Cl7	422.9361	424.9332	C17H24Cl12	650.8003	652.7974

C13H20Cl8	458.8942	456.8971	C17H23Cl13	684.7613	686.7584
C13H19Cl9	492.8552	490.8582			
C13H18Cl10	526.8163	528.8134			
C13H17Cl11	560.7767	562.7737			
C13H16Cl12	594.7377	596.7348			

Table S3. Quantification and qualification ions of LCCPs

Formula	Quantification ion	Qualification ion	Formula	Quantification ion	Qualification ion
C18H34Cl4	389.1336	391.1307	C23H43Cl5	495.1700	497.1670
C18H33Cl5	425.0913	427.0885	C23H42Cl6	529.1310	531.1281
C18H32Cl6	459.0523	461.0495	C23H41Cl7	563.0920	565.0891
C18H31Cl7	493.0133	495.0105	C23H40Cl8	597.0531	599.0501
C18H30Cl8	528.9715	526.9743	C23H39Cl9	631.0141	633.0111
C18H29Cl9	562.9325	560.9354	C23H38Cl10	666.9722	668.9692
C18H28Cl10	596.8935	598.8906	C23H37Cl11	700.9332	702.9302
C18H27Cl11	630.8549	632.8520	C23H36Cl12	734.8942	736.8913
C18H26Cl12	664.8160	666.8130	C23H35Cl13	768.8552	770.8523
C18H25Cl13	698.7770	700.7740	C23H34Cl14	804.8133	806.8104
C18H24Cl14	734.7351	736.7321	C23H33Cl15	838.7744	840.7714
C19H36Cl4	403.1493	405.1463	C24H46Cl4	473.2275	475.2246
C19H35Cl5	439.1069	441.1042	C24H45Cl5	509.1856	511.1827
C19H34Cl6	473.0680	475.0651	C24H44Cl6	543.1467	545.1437
C19H33Cl7	507.0290	509.0261	C24H43Cl7	577.1077	579.1047
C19H32Cl8	542.9871	540.9900	C24H42Cl8	611.0687	613.0658
C19H31Cl9	576.9481	574.9510	C24H41Cl9	645.0297	647.0268
C19H30Cl10	610.9091	612.9063	C24H40Cl10	680.9878	682.9847
C19H29Cl11	644.8706	646.8676	C24H39Cl11	714.9488	716.9459
C19H28Cl12	678.8316	680.8287	C24H38Cl12	748.9099	750.9069
C19H27Cl13	712.7926	714.7897	C24H37Cl13	782.8709	784.8679
C19H26Cl14	748.7507	750.7478	C24H36Cl14	818.8290	820.8260
C20H38Cl4	417.1649	419.1620	C24H35Cl15	852.7900	854.7871
C20H37Cl5	453.1226	455.1199	C25H48Cl4	487.2432	489.2402
C20H36Cl6	487.0836	489.0808	C25H47Cl5	523.2013	525.1983
C20H35Cl7	521.0446	523.0418	C25H46Cl6	557.1623	559.1594
C20H34Cl8	557.0028	555.0056	C25H45Cl7	591.1233	593.1204
C20H33Cl9	590.9638	588.9667	C25H44Cl8	625.0844	627.0814

C20H32Cl10	624.9248	626.922	C25H43Cl9	659.0454	661.0424
C20H31Cl11	658.8862	660.8833	C25H42Cl10	695.0035	697.0005
C20H30Cl12	692.8473	694.8443	C25H41Cl11	728.9645	730.9615
C20H29Cl13	726.8083	728.8053	C25H40Cl12	762.9255	764.9226
C20H28Cl14	762.7664	764.7634	C25H39Cl13	796.8865	798.8836
C21H40Cl4	431.1806	433.1776	C25H38Cl14	832.8446	834.8417
C21H39Cl5	467.1387	469.1357	C25H37Cl15	866.8057	868.8027
C21H38Cl6	501.0997	503.0967	C26H50Cl4	501.2588	503.2559
C21H37Cl7	535.0607	537.0578	C26H49Cl5	537.2169	539.2140
C21H36Cl8	569.0217	571.0188	C26H48Cl6	571.1780	573.1750
C21H35Cl9	602.9828	604.9798	C26H47Cl7	605.1390	607.1360
C21H34Cl10	638.9409	640.9379	C26H46Cl8	639.1000	641.0971
C21H33Cl11	672.9019	674.8989	C26H45Cl9	673.0610	675.0581
C21H32Cl12	706.8629	708.8600	C26H44Cl10	709.0191	711.0162
C21H31Cl13	740.8239	742.8210	C26H43Cl11	742.9801	744.9772
C21H30Cl14	776.7820	778.7791	C26H42Cl12	776.9412	778.9382
C22H42Cl4	445.1962	447.1933	C26H41Cl13	810.9022	812.8992
C22H41Cl5	481.1543	483.1514	C26H40Cl14	846.8603	848.8573
C22H40Cl6	515.1154	517.1124	C27H52Cl4	515.2745	517.2715
C22H39Cl7	549.0764	551.0734	C27H51Cl5	551.2326	553.2296
C22H38Cl8	583.0374	585.0344	C27H50Cl6	585.1936	587.1907
C22H37Cl9	616.9984	618.9955	C27H49Cl7	619.1546	621.1517
C22H36Cl10	652.9565	654.9536	C27H48Cl8	653.1157	655.1127
C22H35Cl11	686.9175	688.9146	C27H47Cl9	687.0767	689.0737
C22H34Cl12	720.8786	722.8756	C27H46Cl10	723.0348	725.0318
C22H33Cl1	754.8396	756.8366	C27H45Cl1	756.9958	758.9928

3			1		
C22H32Cl14	790.7977	792.7947	C27H44Cl12	790.9568	792.9539
C22H31Cl15	824.7587	826.7558	C27H43Cl13	824.9178	826.9149
C23H44Cl4	459.2119	461.2089	C27H42Cl14	860.8759	862.8730
			C27H41Cl15	894.8370	896.8340

Table S4. Parameters used for the estimation of daily intake of CPs.

parameters	adults	toddlers	reference
Average DIR (mg/day)	20	50	(Ali et al. 2013)
Highest DIR (mg/day)	50	100	(Ali et al. 2013)
Exposure fraction (%)	63.8	86.1	(Abdallah and Covaci 2014)
BW (China, kg)	62	9.9	Tan et al. 2019)
BW (United State, kg)	70	12	(Tan et al. 2019)
BSA (m^2)	2.05	0.53	US EPA, 2011
AS (g/ m^2)	0.022	0.022	US EPA, 2011
AF	0.14	0.14	(Wang et al. 2016)
BA (SCCPs, %)	11.7-45.8	11.7-45.8	(Du et al. 2021)
BA (MCCPs, %)	7.9-36.6	7.9-36.6	(Du et al. 2021)
BA (LCCPs, %)	2.6-12.1	2.6-12.1	(Yuan et al. 2021)

Table S5. Concentration of CPs in indoor dust from different regions worldwide.

Sample site	time	Concentration (μg/g)						Analytical instrument	reference
		SCCPs		MCCPs		LCCPs			
		Range	Median	Range	Median	Range	Median		
Guangzhou, China	2015-2016	5.51-158	23.1	11.8-198	36.2	4.22-145	32.8	UPLC-ESI-QTOFMS	This study
Carbondale, America	2017	3.27-107	9.36	9.15-97.2	39.5	2.68-49.5	15.4	UPLC-ESI-QTOFMS	This study
Oslo, Norway	2013-2014	0.76-460	5.8	2.3-840	21	0.66-340	8.1	APCI-Orbitrap-HRMS	(Yuan et al. 2021)
Pretoria, South Africa	2018	2.1-353	16	13-498	46	1.9-108	11	APCI-QTOFMS	(Brits et al. 2020)
Australia	2015	0.29-58	9.4	5.1-530	95	nd.-27	-	APCI-QTOFMS	(He et al. 2019)
Canada [a]	2014	21-65	55	140-192	185	92-161	134	APCI-QTOF-MS	(Wong et al. 2017)
China [a]	2012	106-807	580	330-1948	1398	153-1995	1432	APCI-QTOF-MS	(Wong et al. 2017)
Sweden [a]	2014	4.7-8.8	6.7	66-158	101	567-1263	710	APCI-QTOF-MS	(Wong et al. 2017)
United Kingdom	2008-2009	93	-	463	-	156	-	APCI-QTOF-MS	(Wong et al. 2017)
Australia [a]	2014	61	-	180	-	99	-	APCI-QTOF-MS	(Wong et al. 2017)
Colombian	2016-2019	N/A	40	N/A	73	N/A	13	UPLC-Orbitrap MS	(McGrath et al. 2023)
Australia	2016-2019	N/A	30	N/A	71	N/A	7.7	UPLC-Orbitrap MS	(McGrath et al. 2023)
Thailand	2016-2019	N/A	29	N/A	120	N/A	12	UPLC-Orbitrap MS	(McGrath et al. 2023)
Japan	2016-2019	N/A	22	N/A	30	N/A	3.7	UPLC-Orbitrap MS	(McGrath et al. 2023)
Harbin, China	2013	10.1-173	47.2	-	-	-	-	GC-ECNI-LRMS	(Liu et al. 2017)
Beijing, China	2014-2015	5.35-1022	98.7	2.10-725	89.8	-	-	GC-TOF-HRMS	(Gao et al. 2018)
Qingyuan, China	2016-2017	27.8-173	46.5	74.0-539	166	-	-	GC-ECNI-LRMS	(Chen et al. 2018)
Bavaria, Germany	N/A	nd.-27	5	8-892	176	-	-	GC-ECNI-LRMS	(Hilger et al. 2013)
13 cities, Canada	2007-2010	5.1-353	6.2	5.9-901	19	-	-	GC-ECNI-LRMS	(Shang et al. 2019)

N/A: not available; nd.: not detected; [a] office dust

Table S6. Spearman correlation of SCCPs, MCCPs and LCCPs in house dust from South China and Midwestern U.S.

		**SCCPs**	**MCCPs**	**LCCPs**
South China	SCCPs	1.000		
	MCCPs	0.717***	1.000	
	LCCPs	0.554***	0.726***	1.000
Midwestern U.S.	SCCPs	1.000		
	MCCPs	0.593*	1.000	
	LCCPs	0.554*	0.832***	1.000

* p <0.05; *** p <0.001

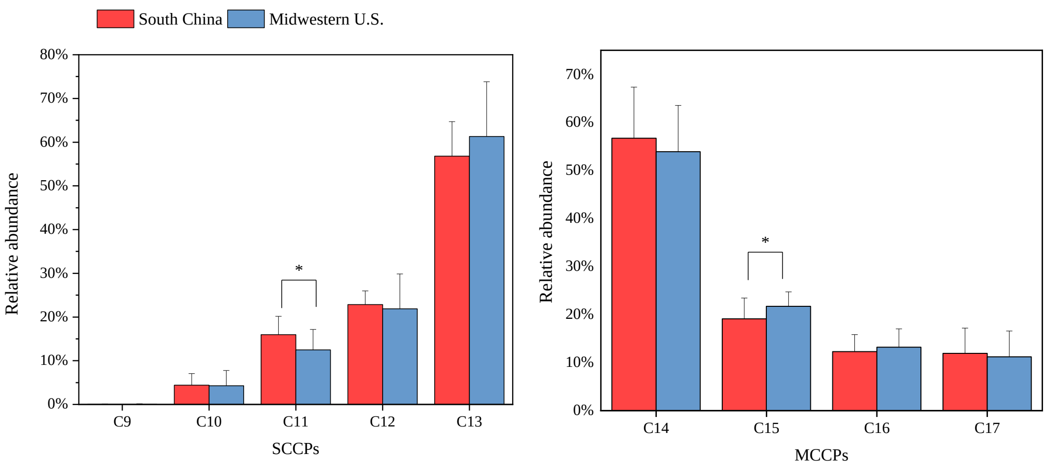

Figure S1. The mean relative abundance of carbon homologues of SCCPs and MCCPs in house dust from South China and Midwestern U.S.

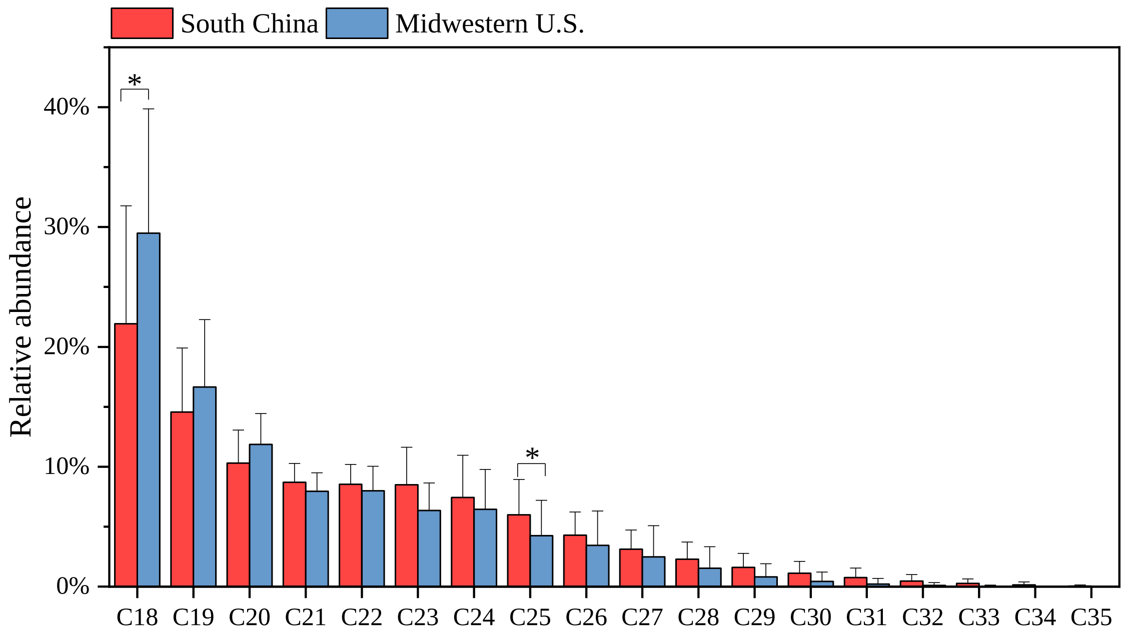

Figure S2. The mean relative abundance of carbon homologues of LCCPs in house dust from South China and Midwestern U.S.

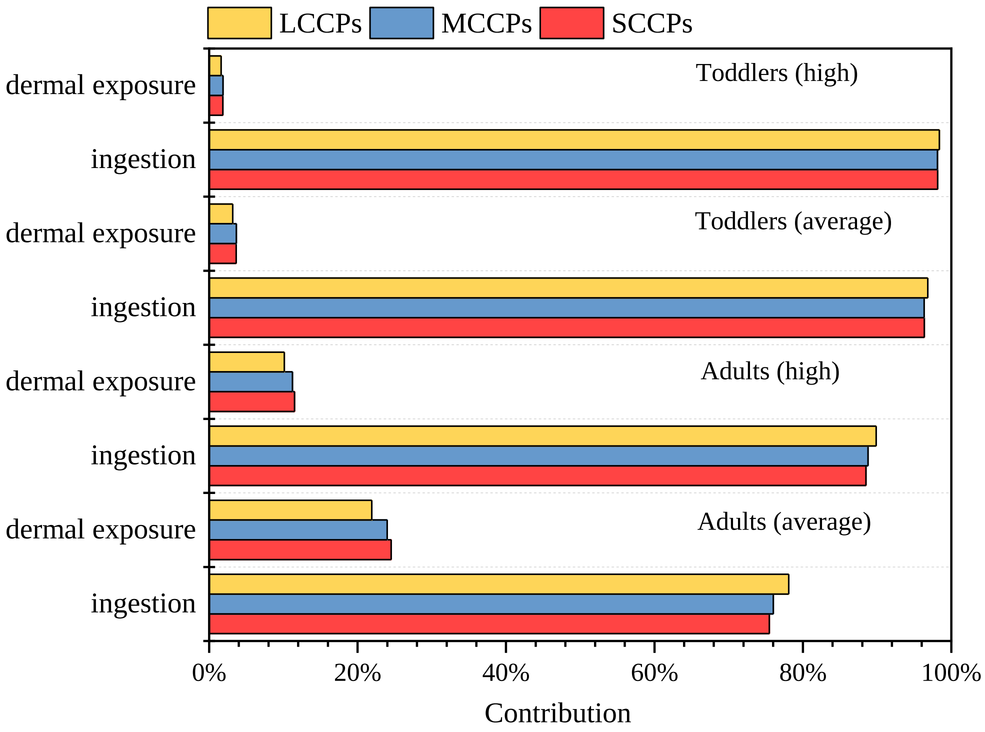

Figure S3. Contribution of estimated daily intakes via dermal exposure and ingestion of dust in toddlers and adults from South China and Midwestern U.S. under different exposure scenarios

References

Abdallah MA, Covaci A. 2014. Organophosphate flame retardants in indoor dust from egypt: Implications for human exposure. Environ Sci Technol 48:4782-4789.

Ali N, Ali L, Mehdi T, Dirtu AC, Al-Shammari F, Neels H, et al. 2013. Levels and profiles of organochlorines and flame retardants in car and house dust from kuwait and pakistan: Implication for human exposure via dust ingestion. Environ Int 55:62-70.

Brits M, de Boer J, Rohwer ER, De Vos J, Weiss JM, Brandsma SH. 2020. Short-, medium-, and long-chain chlorinated paraffins in south african indoor dust and cat hair. Chemosphere 238:124643.

Chen H, Lam JCW, Zhu M, Wang F, Zhou W, Du B, et al. 2018. Combined effects of dust and dietary exposure of occupational workers and local residents to short- and medium-chain chlorinated paraffins in a mega e-waste recycling industrial park in south china. Environ Sci Technol 52:11510-11519.

Du X, Zhou Y, Li J, Wu Y, Zheng Z, Yin G, et al. 2021. Evaluating oral and inhalation bioaccessibility of indoor dust-borne short- and median-chain chlorinated paraffins using in vitro tenax-assisted physiologically based method. J Hazard Mater 402:123449.

Gao W, Cao D, Wang Y, Wu J, Wang Y, Wang Y, et al. 2018. External exposure to short- and medium-chain chlorinated paraffins for the general population in beijing, china. Environ Sci Technol 52:32-39.

He C, Brandsma SH, Jiang H, O'Brien JW, van Mourik LM, Banks AP, et al. 2019. Chlorinated paraffins in indoor dust from australia: Levels, congener patterns and preliminary assessment of human exposure. Sci Total Environ 682:318-323.

Hilger B, Fromme H, Volkel W, Coelhan M. 2013. Occurrence of chlorinated paraffins in house dust samples from bavaria, germany. Environ Pollut 175:16-21.

Liu LH, Ma WL, Liu LY, Huo CY, Li WL, Gao CJ, et al. 2017. Occurrence, sources and human exposure assessment of sccps in indoor dust of northeast china. Environ Pollut 225:232-243.

McGrath TJ, Poma G, Hutinet S, Fujii Y, Dodson RE, Johnson-Restrepo B, et al. 2023. An international investigation of chlorinated paraffin concentrations and homologue distributions in indoor dust. Environ Pollut 333:121994.

Shang H, Fan X, Kubwabo C, Rasmussen PE. 2019. Short-chain and medium-chain chlorinated paraffins in canadian house dust and nist srm 2585. Environ Sci Pollut Res Int 26:7453-7462.

Tan H, Yang L, Yu Y, Guan Q, Liu X, Li L, et al. 2019. Co-existence of organophosphate di- and tri-esters in house dust from south china and midwestern united states: Implications for human exposure. Environ Sci Technol 53:4784-4793.

Wang Y, Hu J, Lin W, Wang N, Li C, Luo P, et al. 2016. Health risk assessment of migrant workers' exposure to polychlorinated biphenyls in air and dust in an e-waste recycling area in china: Indication for a new wealth gap in environmental rights. Environ Int 87:33-41.

Wong F, Suzuki G, Michinaka C, Yuan B, Takigami H, de Wit CA. 2017. Dioxin-like activities, halogenated flame retardants, organophosphate esters and chlorinated

paraffins in dust from australia, the united kingdom, canada, sweden and china. Chemosphere 168:1248-1256.

Yuan B, Tay JH, Padilla-Sanchez JA, Papadopoulou E, Haug LS, de Wit CA. 2021. Human exposure to chlorinated paraffins via inhalation and dust ingestion in a norwegian cohort. Environ Sci Technol 55:1145-1154.

US EPA. Exposure Factors Handbook; US Environmental Protection Agency: Washington, DC, 2011; EPA/600/R-09/052 F.
